# ChCl: Gly (DESs) Promote Environmentally Benign Synthesis of Xanthene Derivatives and Their Antitubercular Activity

**DOI:** 10.3390/molecules26123667

**Published:** 2021-06-16

**Authors:** Mashooq A. Bhat, Ahmed M. Naglah, Siddique Akber Ansari, Hanaa M. Al-Tuwajiria, Abdullah Al-Dhfyan

**Affiliations:** 1 Department of Pharmaceutical Chemistry, College of Pharmacy, King Saud University, Riyadh 11451, Saudi Arabia; ansariakber@gmail.com (S.A.A.); mashooqbhat@rediffmail.com (H.M.A.-T.); 2 Department of Pharmaceutical Chemistry, Drug Exploration and Development Chair (DEDC), College of Pharmacy, King Saud University, Riyadh 11451, Saudi Arabia; anaglah@ksu.edu.sa; 3 Peptide Chemistry Department, Chemical Industries Research Division, National Research Centre, Dokki, Cairo 12622, Egypt; 4 Department of Pharmacology and Toxicology, College of Pharmacy, King Saud University, Riyadh 11451, Saudi Arabia; aaldhfyan@kfshrc.edu.sa; 5 Stem Cell & Tissue Re-Engineering Program, Research Center, King Faisal Specialized Hospital, MBC-03, P.O. Box 3354, Riyadh 11211, Saudi Arabia

**Keywords:** ChCl: Gly, environmentally benign, xanthenes, antitubercular, antibacterial activity, cytotoxicity

## Abstract

A ChCl: Gly (DESs) promoted environmentally benign method was developed for the first time using the reaction of aryl aldehydes and dimedone to give excellent yields of xanthene analogues. The major application of this present protocol is the use of green solvent, a wide range of substrate, short reaction times, ease of recovery, the recyclability of the catalyst, high reaction yield, and ChCl: Gly as an alternative catalyst and solvent. In addition to this, all the synthesized compounds were evaluated for their in vitro antimycobacterial activity against *M. tuberculosis H37Ra (MTB)* and *M. bovis BCG* strains. The compounds **3d**, **3e**, **3f**, and **3j** showed significant antitubercular activity against *MTB* and *M. bovis* strains with minimum inhibitory concentration (MIC) values of 2.5−15.10 µg/mL and 0.26–14.92 µg/mL, respectively. The compounds **3e**, **3f**, and **3j** were found to be nontoxic against MCF-7, A549, HCT 116, and THP-1 cell lines. All the prepared compounds were confirmed by ^1^H NMR and ^13^C NMR analysis.

## 1. Introduction

In the last few years, deep eutectic solvents (DESs) have emerged as ecofriendly and environmentally benign reaction protocols because of the exclusive physicochemical properties, including negligible vapor pressure, nontoxicity, relatively wide liquid range, biodegradability, and bio renewability [1,2]. DESs are easily prepared by the reaction of hydrogen bond donor compounds, such as urea, succinic acid, malonic acid, and zinc chloride with choline chloride as a quaternary ammonium salts. DESs also behave in a different way than their individual components. The melting point of DESs is less than their parent components due to charge delocalization occurring through hydrogen bonding between the hydrogen donor component and halide ion [3]. The glycerol acts as an efficient promoting medium for the electrophilic activation of aldehydes in the reaction [4]. Choline chloride-based DESs have been efficiently used for condensation reactions [5], benzylation of phenol [6], synthesis of spirooxoindole [7], Pictet–Spengler reaction [8], Clauson–Kaas reaction [9], Perkin reaction [10], *N*-alkylation of amines [11], synthesis of pyrroles and furans [8], isolation of residual KOH content from biodiesel [12], deacidification of palm oil [13], synthesis of highly substituted cyclohexa-1,3-dienamines [14], extraction of phenolic compounds from oil mixtures [15], extraction of vanillin [16], and pretreatment of rice straw for butanol fermentation [17].

Tuberculosis is the ninth leading cause of death worldwide from a single infectious agent, ranking above human immune deficiency virus (HIV)/acquired immune deficiency syndrome (AIDS). It is a fatal airborne disease caused by *Mycobacterium tuberculosis* (*Mtb*), which affects the lung and is also responsible for infection in other sites of the body [18]. The global tuberculosis report of 2019 given by the World Health Organization (WHO) shows that TB is the leading cause of deaths due to antimicrobial resistance and among people with HIV [19]. More than half a million new TB cases were notified to WHO by national authorities [20]. Hence, the development of new therapeutic agents against TB is the urgent need of the time.

**Figure 1:**
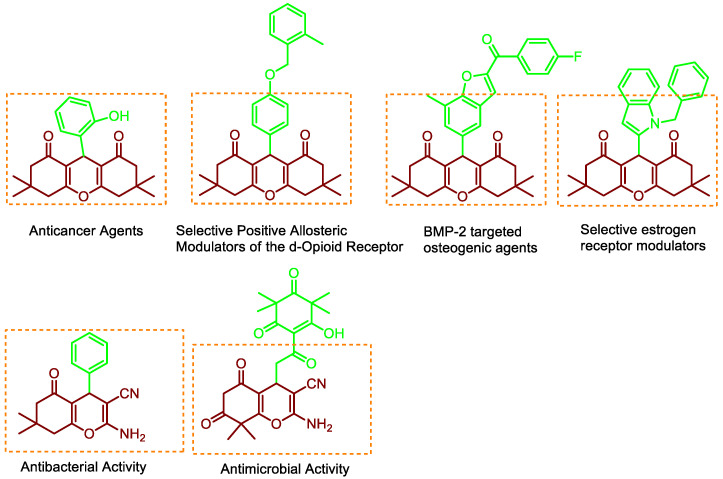
Xanthene-incorporated bioactive molecules.

According to a literature survey, xanthene derivatives are endowed with various biological activities, including potent antimicrobial [21], analgesic [22], antimalarial [23], anti-inflammatory [24], antiviral [25], antioxidant [26], antiproliferative activity [27], anticancer [28], and urease activity [29]; inhibition of trypanothione reductase [23]; bone morphogenetic protein (BMP-2)-targeted osteogenic agents [30]; selective positive allosteric modulators of the δ-opioid receptors [31]; and estrogen receptors [32]. The structures of biologically active xanthene analogues are presented in Figure 1.

The expansion of a synthetic route for the synthesis of these biologically active compounds using a facile, environmentally benign and nontoxic catalyst is of enormous significance from the academic as well as industrial points of view. In the last few years, a large number of protocols have been developed by researchers, including sulfuric acid or hydrochloric acid [33], silica sulfuric acid [34], sulfamic acid [35], *p*-dodecylbenzenesulfonic acid [36], boric acid [37], *p*-toluene sulfonic acid (*p-*TSA) [38], NaHSO_4_-SiO_2_ [39], TiO_2_-SO_4_^2−^ [40], molecular iodine [41], amberlyst-15 [42], and cyanuric chloride [43]. However, a large number of the above routes have some drawbacks, such as the use of hazardous reagents, long reaction times, strong acidic conditions and lower yields of final products. Therefore, there is an urgent need for innovative and environmentally benign protocols to synthesize biologically active compounds with ease. Keeping in mind the pharmacological significance of xanthene derivatives and application of DESs for organic transformation, and in continuation of our work on the synthesis and bio evaluation of numerous compounds [44], herein, we report for the first time choline chloride: glycerol (ChCl: Gly)-based synthesis of xanthene derivatives in excellent yields. The synthesized products were evaluated for their antitubercular, cytotoxicity and antibacterial activities.

## 2. Materials and Methods

### 2.1. Preparation of ChCl: Gly

A mixture of ChCl (7.00 g, 50 mmol) and glycerol (11.65 g, 100 mmol) was added to a round-bottomed flask. The reaction mixture was stirred at 70 °C until the formation of colorless homogeneous liquid. This reaction follows the green chemistry rules [45].

### 2.2. General Procedure for Synthesis of Xanthene Derivatives

A mixture of benzaldehyde **1a** (1 mmol) and dimedone **2** (1 mmol) in ChCl: Gly (2 mL) was stirred at a temperature of 80 °C. The progress of the reaction was monitored by thin-layer chromatography [*n*-hexane/ethyl acetate (3:7)] as a mobile phase. After completion of the reaction, the reaction mass was allowed to cool for 30 min and poured on ice-cold water. Thus, the solid compound obtained was filtered, dried out, and crystallized in ethanol to give the pure product. The synthesized compound was confirmed by m.p., ^1^H NMR, and ^13^C NMR.

#### 2.2.1. 3,3,6,6-Tetramethyl-9-phenyl-3,4,5,6,7,9-hexahydro-1*H*-xanthene-1,8(2*H*)-dione (**3a**)

Compound **3a** was obtained from condensation reaction **1a** and **2** as a white solid; m.p.: 205–206 °C; Yield: 93%; ^1^H NMR (500 MHz, CDCl_3_) δ 11.93 (s, 1H), 7.29–7.26 (t, *J* = 7.6 Hz, 2H), 7.19–7.09 (m, 3H), 5.56 (d, *J* = 60.1 Hz, 1H), 2.49–2.30 (m, 9H), 1.24–1.11 (dd, *J* = 64.4, 29.0 Hz, 12H); ^13^C NMR (101 MHz, CDCl_3_) δ 195.65 (C=O), 164.07 (C=C-O), 143.16 (Ar-H), 135.05 (Ar-H), 129.49 (Ar-H), 127.51 (Ar-H), 119.84 (Ar-H), 50.45 (CH), 42.23 (CH_2_), 32.45 (CH_2_), 31.34 (CH_2_), 29.56 (CH_3_) and 29.33 (CH_3_); High-resolution mass spectroscopy (HRMS) (ESI-qTOF): Calcd for C_23_H_27_O_3_ [M+H]^+^, 351.0920: found: 351.0903.

#### 2.2.2. 3,3,6,6-Tetramethyl-9-(m-tolyl)-3,4,5,6,7,9-hexahydro-1*H*-xanthene-1,8(2*H*)-dione (**3b**)

Compound **3b** was obtained from condensation reaction **1a** and **2** as a white solid; m.p.: 208–210 °C; Yield: 86%; ^1^H NMR (400 MHz, CDCl_3_) δ 7.10–6.99 (m, 3H), 6.86 (d, *J* = 6.8 Hz, 1H), 4.68 (s, 1H), 2.44 (s, 4H), 2.24 (s, 3H), 2.20–2.10 (t, *J* = 13.8 Hz, 4H), 1.05 (s, 6H), 0.95 (s, 6H); ^13^C NMR (101 MHz, CDCl_3_) δ 196.31 (C=O), 162.23 (C=C-O), 144.01 (Ar-H), 137.21 (Ar-H), 129.38 (Ar-H), 127.84 (Ar-H), 127.13 (Ar-H), 125.21 (Ar-H), 115.67 (Ar-H), 50.76 (CH), 40.84 (CH_2_), 32.17 (CH_2_), 31.68 (CH_2_), 29.24 (CH_2_), 27.30 (CH_3_) and 21.48 (CH_3_); High-resolution mass spectroscopy (HRMS) (ESI-qTOF): Calcd for C_24_H_29_O_3_ [M + H]^+^, 365.0650: found: 365.0673.

#### 2.2.3. 3,3,6,6-Tetramethyl-9-(p-tolyl)-3,4,5,6,7,9-hexahydro-1*H*-xanthene-1,8(2*H*)-dione (**3c**)

Compound **3c** was obtained from condensation reaction **1c** and **2** as a yellow solid; m.p.: 222–224 °C; Yield: 90%; ^1^H NMR (400 MHz, CDCl_3_) δ 7.15–7.14 (d, *J* = 7.8 Hz, 2H), 6.99–6.97 (d, *J* = 7.5 Hz, 2H), 4.68 (s, 1H), 2.43 (s, 4H), 2.20 (s, 3H), 2.17–2.09 (m, 4H), 1.06 (s, 6H), 0.95 (s, 6H); ^13^C NMR (101 MHz, CDCl_3_) δ 196.36 (C=O), 162.17 (C=C-O), 141.21(Ar-C), 135.61(Ar-C), 128.71 (Ar-C), 128.20 (Ar-C), 115.67 (Ar-C), 50.75 (CH), 40.81 (CH_2_), 32.13 (CH_2_), 31.40 (CH_2_), 29.23 (CH_2_), 27.31 (CH_3_) and 21.02 (CH_3_); High-resolution mass spectroscopy (HRMS) (ESI-qTOF): Calcd for C_24_H_29_O_3_ [M + H]+, 365.5041: found: 365.5012.

#### 2.2.4. 9-(3-Methoxyphenyl)-3,3,6,6-tetramethyl-3,4,5,6,7,9-hexahydro-1*H*-xanthene-1,8(2*H*)-dione (**3d**)

Compound **3d** was obtained from condensation reaction **1d** and **2** as a yellow solid; m.p.: 162–164 °C; Yield: 84%; ^1^H NMR (400 MHz, CDCl_3_) δ 7.05–7.01 (t, *J* = 8.1 Hz, 1H), 6.80–6.79 (d, *J* = 6.9 Hz, 2H), 6.57–6.55 (d, *J* = 7.0 Hz, 1H), 4.66 (s, 1H), 3.66 (s, 3H), 2.40 (s, 4H), 2.17–2.06 (q, *J* = 16.2 Hz, 4H), 1.01 (s, 6H), 0.91 (s, 6H); ^13^C NMR (101 MHz, CDCl_3_) δ 196.26 (C=O), 162.35 (C=C-O), 159.26 (Ar-C), 145.68 (Ar-C), 128.77 (Ar-C), 120.72 (Ar-C), 115.42 (Ar-C), 114.26 (Ar-C), 111.78 (Ar-C), 55.01(OCH_3_), 50.70 (CH), 40.75 (CH_2_), 32.09 (CH_2_), 31.73 (CH_2_), 29.18 (CH_3_) and 27.29 (CH_3_); High-resolution mass spectroscopy (HRMS) (ESI-qTOF): Calcd for C_24_H_29_O_4_ [M+H]^+^, 381.1631: found: 381.1645.

#### 2.2.5. 9-(4-Methoxyphenyl)-3,3,6,6-tetramethyl-3,4,5,6,7,9-hexahydro-1*H*-xanthene-1,8(2*H*)-dione (**3e**)

Compound **3e** was obtained from condensation reaction **1e** and **2** as a yellow solid; m.p.: 248–250 °C; Yield: 90%; ^1^H NMR (400 MHz, CDCl_3_) δ 7.17–7.15 (m, 2H), 6.71–6.69 (dd, *J* = 5.8, 2.6 Hz, 2H), 4.65 (s, 1H), 3.68 (d, *J* = 4.9 Hz, 3H), 2.42 (s, 4H), 2.20–2.09 (m, 4H), 1.05 (s, 6H), 0.94 (s, 6H); ^13^C NMR (101 MHz, CDCl_3_) δ 196.40 (C=O), 162.05 (C=C-O), 157.90 (Ar-C), 136.47 (Ar-C), 129.25 (Ar-C), 115.72 (Ar-C), 113.41 (Ar-C), 55.05 (CH_3_), 50.74 (CH), 40.81 (CH_2_), 32.14 (CH_2_), 30.92 (CH_2_), 29.23 (CH_3_) and 27.29 (CH_3_); High-resolution mass spectroscopy (HRMS) (ESI-qTOF): Calcd for C_24_H_29_O_4_ [M+H]^+^, 381.1150: found: 381.1132.

#### 2.2.6. 9-(3,4-Dimethoxyphenyl)-3,3,6,6-tetramethyl-3,4,5,6,7,9-hexahydro-1*H*-xanthene-1,8(2*H*)-dione (**3f**)

Compound **3f** was obtained from condensation reaction **1f** and **2** as a yellow solid; m.p.: 200–202 °C; Yield: 89%; ^1^H NMR (400 MHz, CDCl_3_) δ 6.88 (s, 1H), 6.75–6.68 (td, *J* = 8.2, 4.3 Hz, 2H), 4.68 (s, 1H), 3.83 (d, *J* = 1.8 Hz, 3H), 3.77 (d, *J* = 1.7 Hz, 3H), 2.44 (s, 4H), 2.24–2.14 (m, 4H), 1.09 (s, 6H), 0.98 (s, 6H); ^13^C NMR (101 MHz, CDCl_3_) δ 196.42 (C=O), 162.08 (C=C-O), 148.44 (Ar-C), 147.45 (Ar-C), 136.98 (Ar-C), 120.09 (Ar-C), 115.73 (Ar-C), 112.31 (Ar-C), 110.85 (Ar-C), 55.85 (CH_3_), 50.74 (CH), 40.87 (CH_2_), 32.15 (CH_2_), 31.20 (CH_2_), 29.30 (CH_3_) and 27.14 (CH_3_); High-resolution mass spectroscopy (HRMS) (ESI-qTOF): Calcd for C_25_H_31_O_5_ [M+H]^+^, 411.2860: found: 411.2845.

#### 2.2.7. 3,3,6,6-Tetramethyl-9-(3-nitrophenyl)-3,4,5,6,7,9-hexahydro-1*H*-xanthene-1,8(2*H*)-dione (**3g**)

Compound **3g** was obtained from condensation reaction **1g** and **2** as a red solid; m.p.: 172–174 °C; Yield: 82%; ^1^H NMR (400 MHz, CDCl_3_) δ 8.01–7.95 (m, 2H), 7.79–7.77 (d, *J* = 6.7 Hz, 1H), 7.40–7.38 (d, *J* = 7.8 Hz, 1H), 4.81 (s, 1H), 2.49 (s, 4H), 2.25–2.12 (q, *J* = 16.3 Hz, 4H), 1.09 (s, 6H), 0.97 (s, 6H), ^13^C NMR (101 MHz, CDCl_3_) δ 196.30 (C=O), 162.98 (C=C-O), 148.32 (Ar-C), 146.26 (Ar-C), 135.72 (Ar-C), 128.76 (Ar-C), 112.48 (Ar-C), 121.64 (Ar-C), 114.53 (Ar-C), 50.62 (CH), 40.81 (CH_2_), 32.22 (CH_2_), 32.07 (CH_2_), 29.17 (CH_3_)and 27.29 (CH_3_); High-resolution mass spectroscopy (HRMS) (ESI-qTOF): Calcd for C_23_H_26_NO_5_ [M+H]^+^, 396.3451: found: 396.3469.

#### 2.2.8. 9-(3-Iodophenyl)-3,3,6,6-tetramethyl-3,4,5,6,7,9-hexahydro-1*H*-xanthene-1,8(2*H*)-dione (**3h**)

Compound **3h** was obtained from condensation reaction **1h** and **2** as a pale yellow solid; m.p.: 276–278 °C; Yield: 84%; ^1^H NMR (400 MHz, CDCl_3_) δ 7.53 (s, 1H), 7.42–7.41 (d, *J* = 7.8 Hz, 1H),7.32–7.24 (d, *J* = 7.6 Hz, 1H), 6.96–6.92 (t, *J* = 7.8 Hz, 1H), 4.60 (s, 1H), 2.45 (s, 4H), 2.24–2.14 (m, 4H), 1.08 (s, 6H), 0.99 (s, 6H); ^13^C NMR (101 MHz, CDCl_3_) δ 196.21 (C=O), 162.48 (C=C-O), 146.36 (Ar-C), 137.07 (Ar-C), 135.48 (Ar-C), 129.76 (Ar-C), 128.20 (Ar-C), 115.05 (Ar-C), 94.07 (Ar-C), 50.67 (CH), 40.84 (CH_2_), 32.21 (CH_2_), 31.61 (CH_2_), 29.67 (CH_2_), 29.20 (CH_3_) and 27.32 (CH_3_); High-resolution mass spectroscopy (HRMS) (ESI-qTOF): Calcd for C_23_H_26_IO_3_ [M+H]^+^, 477.3570: found: 477.3526.

#### 2.2.9. 9-(4-Bromophenyl)-3,3,6,6-tetramethyl-3,4,5,6,7,9-hexahydro-1*H*-xanthene-1,8(2*H*)-dione (**3i**)

Compound **3i** was obtained from condensation reaction **1i** and **2** as a red solid; m.p.: 238–240 °C; Yield: 86%; ^1^H NMR (400 MHz, CDCl_3_) δ 7.34–7.32 (d, *J* = 6.9 Hz, 2H), 7.18–7.16 (d, *J* = 6.8 Hz, 2H), 4.69 (s, 1H), 2.46 (s, 4H), 2.25–2.14 (q, *J* = 16.4 Hz, 4H), 1.10 (s, 6H), 0.98 (s, 6H); ^13^C NMR (101 MHz, CDCl_3_) δ 196.26 (C=O), 162.41 (C=C-O), 143.18 (Ar-C), 131.09 (Ar-C), 130.13 (Ar-C), 120.18 (Ar-C), 115.14 (Ar-C), 50.65 (CH), 40.81 (CH_2_), 32.15 (CH_2_), 31.52 (CH_2_), 29.21 (CH_3_) and 27.26 (CH_3_); High-resolution mass spectroscopy (HRMS) (ESI-qTOF):Calcd for C_23_H_26_BrO_3_ [M+H]^+^, 430.2450: found: 430.2400.

#### 2.2.10. 9-(4-Chlorophenyl)-3,3,6,6-tetramethyl-3,4,5,6,7,9-hexahydro-1*H*-xanthene-1,8(2*H*)-dione (**3j**)

Compound **3j** was obtained from condensation reaction **1j** and **2** as a yellow solid; m.p.: 238–240 °C; Yield: 90%; ^1^H NMR (400 MHz, CDCl_3_) δ 7.24 (d, *J* = 8.4 Hz, 2H), 7.21–7.14 (m, 2H), 4.71 (s, 1H), 2.47 (s, 4H), 2.19 (q, *J* = 16.3 Hz, 4H), 1.10 (s, 6H), 0.98 (s, 6H); ^13^C NMR (101 MHz, CDCl_3_) δ 196.28 (C=O), 162.44 (C=C-O), 142.70 (Ar-C), 131.93 (Ar-C), 129.74 (Ar-C), 128.13 (Ar-C), 115.18 (Ar-C), 50.66 (CH), 40.79 (CH_2_), 32.14 (CH_2_), 31.43 (CH_2_), 29.21 (CH_3_) and 27.24 (CH_3_); High-resolution mass spectroscopy (HRMS) (ESI-qTOF): Calcd for C_23_H_26_ClO_3_ [M+H]^+^, 385.1165: found: 385.1129.

#### 2.2.11. 9-(4-Hydroxyphenyl)-3,3,6,6-tetramethyl-3,4,5,6,7,9-hexahydro-1*H*-xanthene-1,8(2*H*)-dione (**3l**)

Compound **3l** was obtained from condensation reaction **1l** and **2** as a red solid; m.p.: 248–250 °C; Yield: 83%; ^1^H NMR (400 MHz, CDCl_3_) δ 7.31 (s, 1H), 7.07–7.05 (d, *J* = 7.8 Hz, 2H), 6.55–6.53 (d, *J* = 7.8 Hz, 2H), 4.66 (s, 1H), 2.46 (s, 4H), 2.26–2.16 (q, *J* = 16.4 Hz, 4H), 1.08 (s, 6H), 0.99 (s, 6H). ^13^C NMR (101 MHz, CDCl_3_) δ 197.30 (C=O), 162.45 (C=C-O), 154.70 (Ar-C), 135.47 (Ar-C), 129.29 (Ar-C), 115.85 (Ar-C), 115.23 (Ar-C), 50.72 (CH_2_), 40.81 (CH_2_), 32.23 (CH_2_), 30.93 (CH_2_), 29.11 (CH_3_) and 27.35 (CH_3_); High-resolution mass spectroscopy (HRMS) (ESI-qTOF): Calcd for C_23_H_27_O_4_ [M+H]^+^, 367.0652: found: 367.0673.

#### 2.2.12. 9-Cyclohexyl-3,3,6,6-tetramethyl-3,4,5,6,7,9-hexahydro-1*H*-xanthene-1,8(2*H*)-dione (**3m**)

Compound **3m** was obtained from condensation reaction **1m** and **2** as a white solid; m.p.: 176–178 °C; Yield: 82%; ^1^H NMR (400 MHz, CDCl_3_) δ 7.67 (s, 2H), 5.46 (s, 1H), 3.31 (s, 2H), 2.50 (s, 4H), 2.23 (s, 4H), 1.22 (s, 1H), 1.06–0.85 (d, *J* = 18.0 Hz, 12H); ^13^C NMR (101 MHz, CDCl_3_) δ 193.30 (C=O), 165.54(C=C-O), 111.77 (Ar-C), 50.43 (CH), 40.71 (CH_2_), 34.51 (CH_2_), 33.01 (CH_2_), 31.25 (CH_2_), 29.20 (CH_2_), 27.42 (CH_2_), 25.93 (CH_2_), 24.52 (Cy-C), 23.21 (Cy-C), 23.15 (CH_3_) and 23.07 (CH_3_); High-resolution mass spectroscopy (HRMS) (ESI-qTOF): Calcd for C_23_H_33_O_3_ [M+H]^+^, 357.1081: found: 357.1064.

## 3. Results and Discussion

### 3.1. Chemistry

**Scheme 1:**
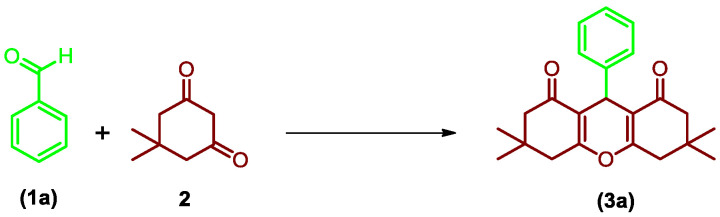
Model reaction.

We studied the model reaction using benzaldehyde (**1a**) (1 mmol), and dimedone (**2**) (1 mmol) for the optimization of the reaction solvents, catalysts, temperatures, and observation of this study is described in Scheme 1 and Table 1.

Initially, we observed that the titled product was not perceived when the model reaction was carried out in the absence of a catalyst (Table 1, Entry 1). In the next step, we tested the reaction using different choline chloride-derived DESs as a reaction medium at 70 °C, which resulted in the desired product **3a** in lower yields, and the results are given in (Table 1, Entry 2–10). In the presence of ChCl: Gly, this results in the formation of compound **3a** in a good yield of 93% at 80 °C for 45 min. It was observed that ChCl: Gly (1:2) was a pre-eminent catalyst for this conversion. According to these results, the catalyst plays an important role in this transformation. We studied several DES catalysts. Among them, ChCl: Gly was highly active due to its relatively low viscosity and high thermal stability [46]. The remaining DESs showed lower activity because of direct interactions of the reactants with DESs, variation in acidity, and the physical properties of DESs. Due to these main reasons, the smooth interaction between reactants was not possible. The ChCl: Gly DES exhibited self-association of glycerol and choline chloride through the interaction of hydrogen bonding, which is the main reason for its high catalytic efficiency.

In the next step, we also screened the effect of temperature on the model reaction. The model reaction was stirred at a different temperature ranges from 70 to 90 °C, with an increment of 10 °C each time. The results exhibited that an increase in the temperature of the model reaction increased the product of the reaction. All these observations suggest that 80 °C is an optimum temperature in terms of reaction time and yield (Table 1, Entries 11, 13–14).

**Scheme 2:**
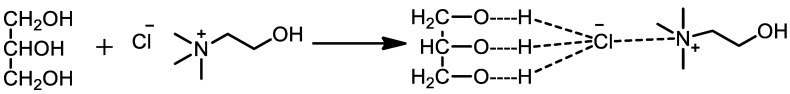
Hydrogen bonding between glycerol and choline chloride.

When the reaction was performed with the help of the glycerol, results in a 62% yield of the titled product as shown in (Table 1, Entry 15). Whereas when the model reaction was performed using the ChCl, results decrease in the yield of titles compound **3a** at 80 °C was shown in (Table 1, Entry 16). This result proves that the excellent yield of the final product is due to the ChCl: Gly (1:2) and not to its individual components, glycerol and choline chloride. This is because of extensive hydrogen bonding between glycerol and quaternary ammonium salt choline chloride (Scheme 2) [46].

Furthermore, we also investigated the catalyst loading on the model reactions. The model reaction was carried out at different catalyst concentrations of 0.5, 1.0, 1.5, 2.0, and 2.5 g of ChCl: Gly (1:2) at 80 °C. Better results were observed when the model reaction was carried out using 2.0 g of ChCl: Gly (1:2) to give 93% yields of the titled product (Table 2, Entry 4).

The recycling and reuse of ChCl: Gly is the main advantage of our planned method. We studied the reusability of the ChCl: Gly. The reaction was carried out using aldehyde and dimedone under the optimized reaction parameters.

After the finishing point of the reaction, the reaction mass was poured on ice-cold water and product was extracted with EtOAc (2 × 20 mL) and an organic layer was washed with 5% NaHCO_3_ then dried over anhydrous NaSO_4_. In this reaction, ChCl: Gly (1:2) was recovered by simply evaporating water from the reaction mass. The recyclability of the catalytic efficiency of ChCl: Gly was tested for five consecutive cycles. There was no significant decrease in the yield observed as shown in Figure 1 (Table 3).

**Figure 2:**
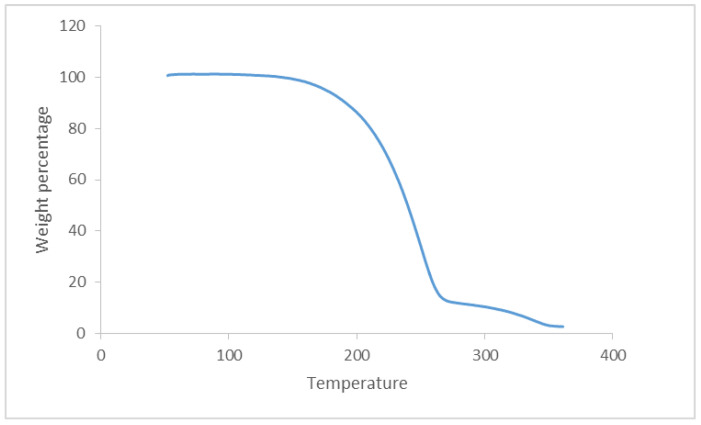
Thermo-gravimetric analysis of ChCl: Gly (DES).

ChCl: Gly (1:2) exhibited prominent catalytic activity with extra thermal stability. Zhao S. H. et al. and Gary A. Baker described the thermal strength of ChCl: Gly at 150 °C [47]. We also confirmed this thermo-gravimetric analysis in a nitrogen atmosphere at 250 °C min^−1^ (Figure 2). This study suggests that the ChCl: Gly is stable up to 150 °C. In the next step, ChCl: Gly is heated at 100 °C for 300 min, resulting in no weight loss being observed. According to these results, our synthesized ChCl: Gly is stable below 100 °C.

**Figure 3:**
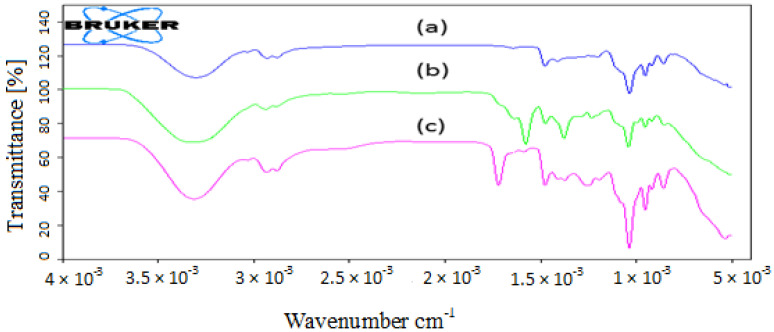
FTIR spectra of (**a**) ChCl: Gly measured before reaction (**b**) ChCl: Gly measured after fifth cycle during recyclability of reaction (**c**) ChCl: Gly measured after 300 min at 100 °C.

We studied the stability of ChCl: Gly during recyclability of **3a** with the help of IR of the recovered ChCl: Gly. The Fourier-transform infrared spectroscopy (FTIR) spectrum of pure ChCl: Gly showed peaks at 1040, 1482, and 2950 cm^−1^ and a broad peak at around 3324 cm^−1^. There was no considerable change in the Fourier-transform infrared spectroscopy (FTIR) spectra of pure ChCl: Gly and the IR spectra of ChCl: Gly recovered after the fifth cycle (Figure 3).

We undertook a comparative study of the ChCl: Gly with other previously reported catalysts for the synthesis of xanthene analogues. The comparison results proved that ChCl: Gly is the better catalyst in terms of its excellent yield and reusability with less reaction time (Table 4, Entry 10). In conclusion, ChCl: Gly is an efficient and greener approach for the synthesis of xanthene analogues.

Compound **3a** was confirmed by ^1^H NMR and ^13^C NMR analysis. In ^1^H NMR spectra of the compound, **3a** shows two distinct singlets at δ 1.21 and 1.24 ppm for the two methyl groups. Peaks were observed at δ 2.49 and 2.30 ppm for the -CH_2_-C=O and CH_2_ protons, respectively. These two signals confirmed that a dimedone ring is present in our synthesized compound. The peak observed at δ 5.56 ppm is due to the presence of -CH proton in compound **3a**. In the ^13^C NMR spectrum of compound **3a**, the peak at δ 195.65 ppm shows the presence of a -C=O group. The methine, methylene, and methane peaks observed at δ 50.43, 42.23, 32.45, 31.34, 29.56, and 29.33 ppm suggest the formation of the final compound **3a**.

**Scheme 3:**
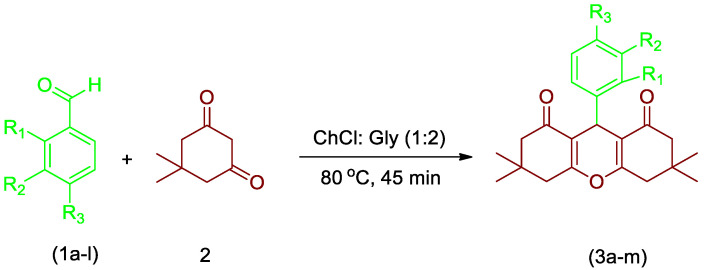

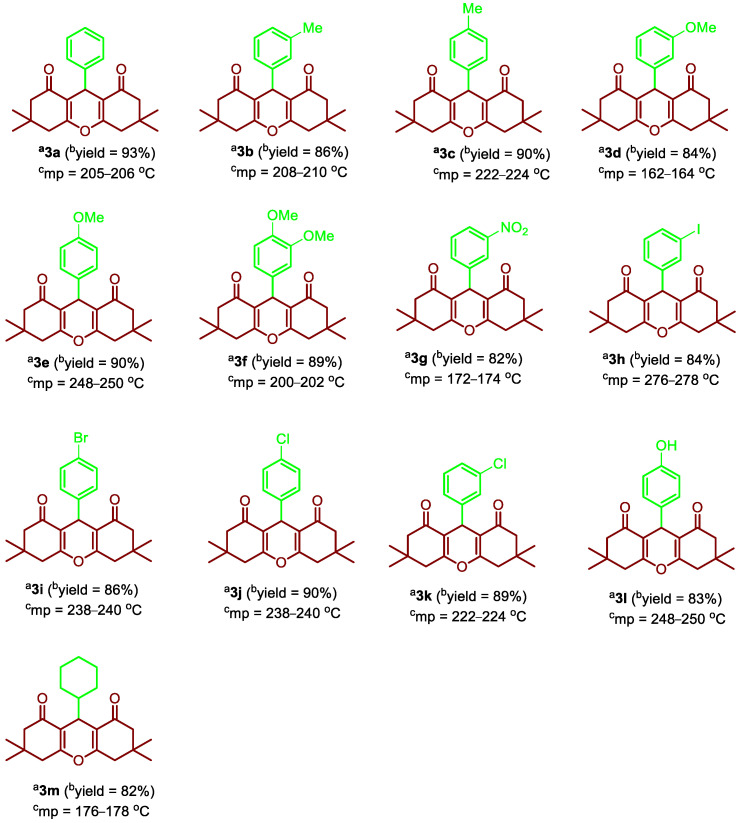
Synthesis of xanthene derivatives (**3a–l**) by using ChCl: Gly as a catalyst. ^a^ Reaction conditions: aldehydes (**1a–m**) (1 mmol) and dimedone (**2**) (1 mmol) in ChCl: Gly (2.0 g) stirred at 45 °C; ^b^ isolated yields, ^c^melting points are in good agreements with previously reported compounds in the literature [50].

In conclusion, the effectiveness and better reaction time for the model reaction was observed at 80 °C by using 2 g of ChCl: Gly as a catalyst. With excellent reaction conditions in hand, the adaptableness of this approach was employing the synthesis xanthene analogues (**3a–l**). Various substituents on aryl aldehyde, including methoxy, methyl, nitro, halogen (-Cl,-Br, -I), and hydroxyl groups, were used. The synthesis of the compounds (**3b–l**) using the optimized reactions conditions and results are shown in Scheme 3. The result clearly suggests that the condensation reactions using ChCl: Gly catalyst show an excellent and remarkable performance irrespective of the electron-withdrawing/-donating groups present on the aryl aldehydes (Table 2), hence this method is facile, efficient, and general for the synthesis of xanthene analogues. All the synthesized final compounds (**3a–l**) were well characterized by ^1^H NMR and ^13^C NMR spectroscopic techniques and are incorporated in the Supplementary Material.

**Scheme 4:**
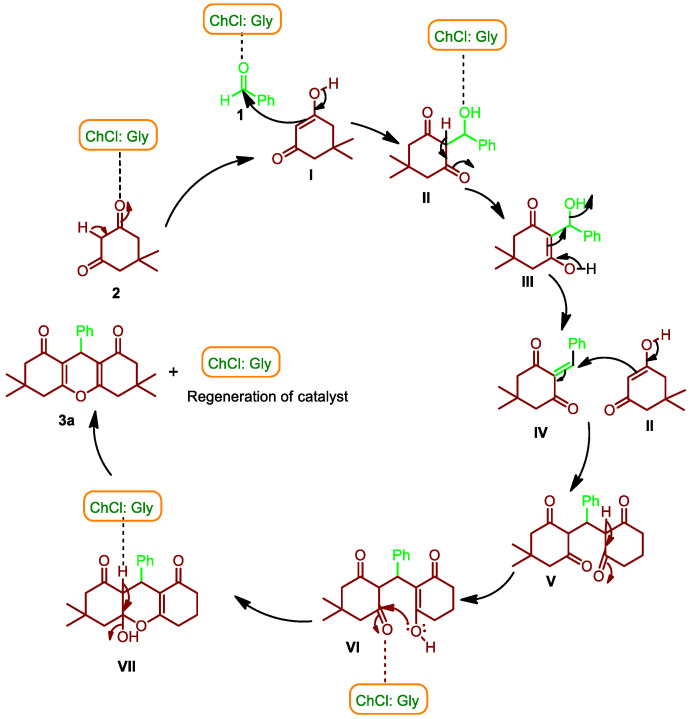
Reaction mechanism cycle for the preparation of compounds **3a**.

The reaction mechanism cycle for the preparation of xanthene analogues employing ChCl: Gly is the catalyst. In the first step, benzaldehyde activated by ChCl: Gly results in the formation of intermediate **I**. In the next step, ChCl: Gly reacts with dimedone to give the enol product **II**. In the third step, intermediate **I** reacts with **II** to afford the addition of the product **III**. Further formation of the alkylation product **V** occurs due to the reaction of **II** and **III** via removal of the H_2_O molecule. In the next step, intramolecular cyclization of **V** occurs to give **VI**. In the last step, elimination of the H_2_O molecule using ChCl: Gly results in the formation of the titled xanthene analogues **3a** and regeneration of the catalyst. The detailed reaction mechanism is presented in Scheme 4.

### 3.2. Biological Activity

#### 3.2.1. Antitubercular Activity Screening

The synthesized xanthene derivatives (**3a–m**) were screened for their in vitro antitubercular activity against *MTB H37Ra* American Type Culture Collection 25177 (ATCC 25177) and *M. bovis BCG* (ATCC 35743) strain in liquid medium [56]. We developed the well-known (2,3-Bis-(2-Methoxy-4-Nitro-5-Sulfophenyl)-2H-Tetrazolium-5-Carboxanilide) (XTT) reduction menadione assay (XRMA) of an antitubercular screening protocol (incorporated in the Supplementary Material) using a standard reference, i.e., rifampicin drug, and the minimum inhibitory concentration (MIC) and inhibitory concentration (IC_50_) values are presented in Table 5.

Among all the synthesized xanthene derivatives compounds, **3d**, **3e**, **3f**, **3j**, and **3k** were found to have prominent antimycobacterial activity against the *MTB H37Ra* and *M. bovis BCG* strain with MIC values of 2.5–15.10 and 0.26–14.92 µg/mL, respectively. However, the rest of the xanthene derivatives **3a**, **3b**, **3c**, **3g**, **3h**, **3i**, **3k**, and **3l** were inactive against the *MTB H37Ra* and *M. bovis BCG* strain with MIC ≥ 30 µg/mL.

#### 3.2.2. Structure–Activity Relationship (SAR)

According to the activity data, the xanthene analogues showed excellent antitubercular activity and the results are presented in Table 5. The biological activity results revealed that the activity was significantly impacted by various substituents present on the aryl ring.

Firstly, we will elaborate the antitubercular activity of xanthene analogues against the *MTB H37Ra* strain. From the xanthene analogues series (**3a–m**), compound **3a**, without any substitution on the aryl ring, displayed lesser potency against the *MTB H37Ra* strain with an MIC value >30 µg/mL as compared to the standard drug rifampicin and the results are displayed in Table 5. Compounds **3b** (R_2_ = *-methyl*) and **3c** (R_3_ = *-methyl*) showed lower antitubercular activity against the *MTB H37Ra* strain with MIC ≥ 30 µg/mL. When the *-methoxy* group was introduced in compound **3d** (R_2_ = *-methoxy*), it showed excellent activity against the *MTB H37Ra* strain with MIC = 15.10 μg/mL. When a *-methoxy* group was present in the aryl ring at the *para* position as in compound **3e** (R_3_ = *-methoxy),* it showed excellent activity against the *MTB H37Ra* strain with an MIC value of 4.20 µg/mL with reference to the standard drug. When a *-methoxy* group was introduced at both R_2_ and R_3_ in compound **3f,** it exhibited prominent antimycobacterial activity against the *MTB H37Ra* strain with MIC = 2.25 μg/mL.

When a *nitro* group was introduced in compound **3g** (R_2_ = -NO_2_), it exhibited less activity against the *MTB H37Ra* strain with MIC ≥ 30 µg/mL with reference to the standard drug. Replacing the *nitro* group with an *iodo* group in compound **3h** (R_2_ = *-I*) resulted in inactivity against the *MTB H37Ra* strain with MIC ≥ 30 µg/mL. Installation of a *bromo* group at the para position of the aryl group in **3i** (R_3_ = -Br) resulted in inactivity against the *MTB H37Ra* strain with MIC ≥ 30 µg/mL. Replacing a *bromo* by a chloro group at the para position in compound **3j** (R_3_ = -Cl) resulted in the compound being highly potent against the *MTB H37Ra* strain with MIC = 4.74 µg/mL. When the position of the *chloro* group was changed in **3k** (R_2_ = -Cl), it showed moderate antitubercular activity against the *MTB H37Ra* strain with MIC = 8.34 µg/mL as compared to compound **3j**. When a *hydroxy* group was present at the para position in compound **3l** (R_3_ = -OH), it was inactive against the *MTB H37Ra* strain with MIC ≥ 30 µg/mL. In compound **3m**, when the aryl ring was replaced by a cyclohexane ring, it was found to be inactive against the *MTB H37Ra* strain with MIC ≥ 30 µg/mL. Hence, among all the synthesized xanthene derivatives **3a–m**, compounds **3d**, **3e**, **3f**, **3j**, and **3k** were found to be highly potent against the *MTB H37Ra* strain and the details are disclosed in Table 5.

Further, all the compounds were also tested for antitubercular activity against the *M. bovis BCG* strain. From the series (**3a–m**), compound **3a**, without any substituent present on the aryl ring, showed no activity against the *M. bovis BCG* strain with MIC ≥ 30 µg/mL and the results are presented in Table 5. Compounds **3b i** (R_2_ = -Me) and **3c** (R_3_ = -Me) were found to be inactive against the *M. bovis BCG* strain with MIC ≥ 30 µg/mL. When *methoxy* was introduced in compound **3d** (R_2_ = -OMe), it presented prominent antitubercular activity against the *M. bovis BCG* strain MIC = 14.92 µg/mL. Introduction of a *methoxy* group at the *para* position of the aryl ring in compound **3e** (R_3_ = -OMe) and **3f** (R_2_ = R_3_ = -OMe) resulted in potency against the *M. bovis BCG* strain with MIC = 0.26 and 1.22 µg/mL, respectively. When a *nitro* group was present at the R_2_ position in compound **3g** (R_2_ = -NO_2_), it was found to be inactive against the *M. bovis BCG* strain with MIC ≥ 30 µg/mL.

Replacing the *nitro* group by an *iodo* group in **3h** (R_2_ = -I), the compound was found to be inactive towards the *M. bovis BCG* strain with MIC ≥ 30 µg/mL. Introduction of a *bromo* group at the para position of the aryl group in **3i** (R_3_ = -Br) resulted in the compound being inactive against the *M. bovis BCG* strain with MIC ≥ 30 µg/mL. When the *chloro* group was present at the *para* position in **3j** (R_3_ = -Cl), the compound was highly active against the *M. bovis BCG* strain with MIC = 2.58 µg/mL. When the position of the *chloro* group was changed in **3k** (R_2_ = -Cl), it showed moderate antitubercular activity against the *M. bovis BCG* strain with MIC = 6.13 µg/mL as compared to compound **3j**. Compound **3l** (R_3_ = *-*OH) did not show antitubercular activity against the *M. bovis BCG* strain with MIC ≥ 30 µg/mL. In compound **3m**, the aryl group was replaced by a cyclohexane ring and the antitubercular activity against the *M. bovis BCG* strain was decreased, with an MIC value >30 µg/mL. Hence, among all the xanthene derivatives (**3a–m**), compounds **3d**, **3e**, **3f**, **3k**, and **3l** were highly potent against the *M. bovis BCG* strain and the results are presented in Table 5.

### 3.3. Cytotoxicity

Among the prepared xanthene analogues, the most active compounds **3d**, **3e**, **3f**, **3j**, and **3k** were further evaluated for cytotoxicity against human cell lines, including MCF-7, A549, HCT 116, and THP-1, using the well-developed tetrazolium salt (3-(4,5-dimethylthiazol-2-yl)-2,5-diphenyltetrazolium bromide (MTT) assay [57]. The toxicity results revealed that most of these xanthene analogues were nontoxic and showed specific activity against the *MTB* and *M. bovis BCG* strains, with GI_50_/GI_90_ (>100 µg/mL) (Table 6).

### 3.4. Selectivity Index (SI)

SI determines the most active compounds selective towards mycobacteria but remain nontoxic against host human cancer cell lines. The drug susceptibility study indicates that when the SI was >10, then these compounds were more selective as antitubercular agents. Therefore, the most active compounds **3e** (SI = 20), **3f** (SI = 44), **3j** (SI = 18), and **3k** (SI = 14) exhibited >10 SI, revealing that these xanthene analogues act as excellent antitubercular agents. The detailed study is summarized in Table 7.

### 3.5. Antibacterial Activity

The specificity of the most active xanthene analogues **3d**, **3e**, **3f**, **3j**, and **3k** was estimated for their antibacterial activity against the Gram-negative bacteria *Escherichia coli* and *Pseudomonas* *fluorescence* and the Gram-positive bacteria *Staphylococcus aureus* and *Bacillus* *subtilis.* According to the antibacterial activity data, the most potent compounds were inactive towards the tested bacterial strains. This activity results show that the most potent compounds were highly specific against the *MTB H37Ra* and *M. bovis BCG* strains and the detailed study is described in Table 8.

### 3.6. ADME Properties

The success of a drug is determined not only by good efficacy but also by an acceptable ADME (absorption, distribution, metabolism, and excretion) profile. In this study, we calculated the molecular volume (MV), molecular weight (MW), logarithm of the partition coefficient (miLog*P*), number of hydrogen bond acceptors (n-ON), number of hydrogen bond donors (n-OHNH), topological polar surface area (TPSA), number of rotatable bonds (n-ROTB), and Lipinski’s rule of five [58] using the Molinspiration online property calculation toolkit [59]. Absorption (% ABS) was calculated by: % ABS = 109 − (0.345 × TPSA) [60].

The drug-likeness model score (a collective property of the physicochemical properties, pharmacokinetics, and pharmacodynamics of a compound represented by a numerical value) was computed by MolSoft software [61]. A computational study of all the synthesized compounds was performed for the prediction of ADME properties and the values obtained are presented in Table 9. It was observed that the compounds exhibited a good % ABS (% absorption), ranging from 69.11 to 85.02%.

Furthermore, the compounds **3j** and **3k** did not violate Lipinski’s rule of five (miLog*P ≤* 5). A molecule that is likely to be developed as an orally active drug candidate should show no more than one violation of the following four criteria: miLog*P* (octanol-water partition coefficient) ≤ 5, molecular weight ≤ 500, number of hydrogen bond acceptors ≤ 10, and number of hydrogen bond donors ≤ 5 [62]. The larger the value of the drug-likeness model score, the higher the probability that the particular molecule will be active. All the tested compounds followed the criteria for orally active drugs and therefore, these compounds may have good potential for eventual development as oral agents.

## 4. Conclusions

In conclusion, a facile and environmentally benign approach was developed for the synthesis of xanthene analogues using a recyclable and inexpensive ChCl: Gly catalytic solvent-free system. This reaction protocol revealed several advantages, including high atom efficiency, mild reaction conditions, uniqueness, easy workup procedure, clean reaction profiles, and eco friendliness. Additionally, all the prepared xanthene analogues were screened for their in vitro antitubercular activity against the *MTB H37Ra* and *M. bovis* *BCG* strains. Among all the screened compounds, **3d**, **3e**, **3f**, **3j**, and **3k** were found to be highly active compounds, with MIC values in the range of 2.5−15.10 µg/mL against the *M. bovis* *BCG* strain and 0.26–14.92 µg/mL against the *MTB* *H37Ra* strain, respectively. The most active compounds were found to be nontoxic against MCF-7, A549, HCT 116, and THP-1 cancer cell lines by employing the MTT method. Highly potent xanthene derivatives **3d**, **3e**, **3f**, **3j**, and **3k** were found to have a selectivity index of >10 against MCF-7, A549, HCT 116, and THP-1, which suggests that they can act as promising antitubercular agents. All these results confirm that compounds **3d**, **3e**, **3f**, **3j**, and **3k** can be further modified as lead drug molecules.

## Figures and Tables

**Table 1:** Optimization of the reaction conditions ^a^.

Entry	Solvent	Temperature (°C)	Time (min)	Yield (%) ^b^
1	-	70	520	-
2	ChCl:Urea(1:2)	70	80	60
3	ChCl:Thiourea(1:2)	70	80	58
5	ChCl:Adipic Acid (1:1)	70	80	28
4	ChCl:Oxalic Acid(1:1)	70	80	63
7	ChCl:Malonic Acid(1:1)	70	80	38
6	ChCl:Succinic Acid(1:1)	70	80	46
9	ChCl:ZnCl_2_ (1:2)	70	85	30
8	ChCl:FeCl_3_ (1:2)	70	85	35
10	ChCl:Gly (1:1)	70	80	76
11	ChCl:Gly (1:2)	70	60	82
12	ChCl:Gly (1:3)	70	60	83
13	ChCl:Gly (1:2)	80	45	93
14	ChCl:Gly (1:2)	90	45	93
15	Gly	80	80	62
16	ChCl	80	520	25

^a^ Reaction conditions: benzaldehyde **1** (1 mmol), dimedone **2** (1 mmol and DES (2.0 g); ^b^ Isolated yields. ChCl: Choline Chloride; Gly: Glycerol.

**Table 2:** Optimization of deep eutectic solvent ^a^.

Entry	ChCl: Gly (g)	Time (min)	Temp (°C)	% Yield ^b^
1	0.5	45	80	28
2	1	45	80	58
3	1.5	45	80	78
4	2	45	80	93
5	2.5	45	80	94

^a^ Reaction conditions: benzaldehyde **1** (1 mmol), dimedone **2** (1 mmol) and ChCl: Gly (2.0 g); ^b^ Isolated yields.

**Table 3:** Recycling of ChCl: Gly (DES) for the synthesis of compound **3a** ^a^.

Entry	Run	Time (min)	% Yield ^b^
1	Fresh	45	93
2	1	45	93
3	2	45	91
4	3	45	89
5	4	45	85
6	5	45	82

^a^ Reaction conditions: benzaldehyde **1** (1 mmol), dimedone **2** (1 mmol) and ChCl: Gly (2.0 g); ^b^ Isolated yields.

**Table 4:** Comparative catalytic study of ChCl: Gly with the reported catalysts.

Entry	Catalyst	Amount of Catalyst	Time (min)	Yield (%)	Solvent/Condition	Ref.
1	*β*-CD (1 mmol)	1 mmol	10 h	96	EtOH/60 °C	[48]
2	SBSSA	30 mg	10 h	98	EtOH/reflux	[49]
3	FeCl_3_.6H_2_O (10 mol%)	10 mol%	6 h	92	[bmim][BF_4_]/80 °C	[50]
4	Amberlyst-15	200 mg	5 h	92	CH_3_CN/Reflux	[51]
5	Cellulose sulfonic acid	50 mg	5 h	94	Solvent free/110 °C	[52]
6	[bmim][HSO_4_] (42 mol%)	42 mol%	3 h	85	Solvent free/100 °C	[40]
7	Fe^3+^ montmorillonite	85 mg	6 h	94	EtOH/reflux	[53]
8	Zr(DP)_2_	10 mol %	24 h	98	EtOH/80 °C	[54]
9	ZnO-CH_3_COCl	30 mol %	5 h	86	CH_3_CN/reflux	[55]
10	ChCl:Gly	2.0 g	45 min	93	ChCl:Gly act as a solvent	Present work

**Table 5:** In vitro antimycobacterial activity of xanthene conjugates in (µg/mL).

Compound	*MTB* H37Ra		*M. bovis* BCG	
	^a^ MIC	^a^ IC_50_	^a^ MIC	^a^ IC_50_
**3a**	>30	>30	>30	>30
**3b**	>30	>30	>30	>30
**3c**	>30	>30	>30	>30
**3d**	15.10	3.20	14.92	3.40
**3e**	4.20	0.74	0.26	0.28
**3f**	2.5	0.79	1.22	0.32
**3g**	>30	>30	>30	>30
**3h**	>30	>30	>30	>30
**3i**	>30	>30	>30	>30
**3j**	4.74	0.50	2.58	0.6
**3k**	7.8	2.3	9.4	5.8
**3l**	>30	>30	>30	>30
**3m**	>30	>30	>30	>30
**^b^** **Rifampicin**	0.045	0.0017	0.017	0.0015

^a^ MIC/IC_50_ in mg/mL. Antitubercular activity of all compounds was investigated using serial dose-dependent dilutions protocol. ^b^ Rifampicin as a standard drug acts as a positive control.

**Table 6:** In vitro cytotoxicity of active xanthene analogues (GI_50_ in μg/mL).

Compound	MCF-7	A549	HCT 116	THP-1
**3d**	>100	>100	>100	>100
**3e**	>100	>100	>100	>100
**3f**	>100	>100	>100	>100
**3j**	>100	>100	>100	>100
**3k**	>100	>100	>100	>100
**Rifampicin**	>100	>100	>100	>100
**Paclitaxel**	0.0048	0.0035	0.0260	0.1374

GI_50_ indicates the concentration that inhibits 50% of the growth of cells.

**Table 7:** Selectivity index against dormant *MTB H37Ra* and *M. bovis BCG*.

Compound	MCF-7		A549		HCT 116		THP-1	
	*MTB*	BCG	*MTB*	BCG	*MTB*	BCG	*MTB*	BCG
**3d**	7	7	7	7	7	7	7	7
**3e**	20	330	20	333	20	330	20	330
**3f**	44	82	42	82	42	82	44	82
**3j**	18	40	18	40	18	40	18	40
**3k**	14	12	14	12	14	12	14	12
**Rifampicin**	196	222	196	222	196	222	196	222

**Table 8:** Antibacterial activity of compounds MIC (μg/mL).

Compound	*E. coli*	*P. fluorescens*	*S. aureus*	*B. subtilis*
**3d**	>30	>30	>30	>30
**3e**	>30	>30	>30	>30
**3f**	>30	>30	>30	>30
**3j**	>30	>30	>30	>30
**3k**	>30	>30	>30	>30
**Kanamycin**	3.34 ± 0.41	1.01 ± 0.09	>61.92	2.78 ± 0.85
**Ampicillin**	4.17 ± 1.04	12.47 ± 1.28	2.86 ± 0.78	29.53 ± 1.88

**Table 9:** Pharmacokinetic parameters important for good oral bioavailability and its drug likeness model score.

Com	% ABS	TPSA (A^2^)	n-ROTB	MV	MW	miLog*P*	n-ON	n-OHNH	Lipinski Violation	Drug Likeness Model Score
Rule	-	-	-	-	<500	≤5	<10	<5	≤1	
**3a**	94.03	43.38	1	336.59	350.46	5.20	3	0	1	−1.31
**3b**	94.03	43.38	1	353.15	364.49	5.62	3	0	1	−0.98
**3c**	94.03	43.38	2	353.15	364.49	5.65	3	0	1	−1.27
**3d**	90.84	52.61	2	362.13	380.48	5.23	4	0	2	−0.89
**3e**	90.84	52.61	3	362.13	380.48	5.25	4	0	2	−1.09
**3f**	87.66	61.84	2	387.8	410.51	4.84	5	0	3	0.41
**3g**	78.22	89.20	1	359.92	395.45	5.13	6	0	1	−0.85
**3h**	94.03	43.38	1	360.58	476.35	6.26	3	0	1	−0.87
**3i**	94.03	43.38	1	354.48	429.35	6.01	3	0	1	−1.16
**3j**	94.03	43.38	1	350.12	384.90	5.88	3	0	1	−0.77
**3k**	87.05	63.60	1	344.61	366.46	4.72	4	0	1	−0.72
**3l**	94.03	43.38	1	355.18	356.51	5.79	3	1	1	−1.32

Com: Compound, % ABS: percentage absorption, TPSA: topological polar surface area, n-ROTB: number of rotatable bonds, MV: molecular volume, MW: molecular weight, milogP: logarithm of partition coefficient of the compound between n-octanol and water, n-ON acceptors: number of hydrogen bond acceptors, n-OHNH donors: number of hydrogen bonds donors.

## Data Availability

Biological activity protocol and supplementary data (copies of ^1^H and ^13^C NMR spectra of all the synthesized compounds) associated with this article can be found through the “Supplemental Content” section of this article’s webpage.

## Supplementary Materials

The following are available online. Biological activity protocol and supplementary data (copies of ^1^H and ^13^C NMR spectra of all the synthesized compounds).

## Sample Availability

Samples of the compounds are available from authors.
