# Supplementary material for: ChCl: Gly (DESs) Promote Environmentally Benign Synthesis of Xanthene Derivatives and Their Antitubercular Activity
Source: Molecules. 2021 Jun 16;26(12):3667. doi: 10.3390/molecules26123667 (PMC8235460; doi:10.3390/molecules26123667)

ChCl:Gly (DESs) Promoted Environmentally Benign Synthesis of Xanthene Derivatives and Their Antitubercular Activity

Mashooq A. Bhat[a,*], Ahmed M. Naglah[b,c], Siddique Akber Ansari[a], Hanaa M. Al-Tuwajiria[a], Abdullah Al-Dhfyan[d,e]

[a]*Department of Pharmaceutical Chemistry, College of Pharmacy, King Saud University, Riyadh 11451, Saudi Arabia*

[b]*Department of Pharmaceutical Chemistry, Drug Exploration and Development Chair (DEDC), College of Pharmacy, King Saud University, Riyadh 11451, Saudi Arabia*

[c]*Peptide Chemistry Department, Chemical Industries Research Division, National Research, Centre, Dokki, Cairo-12622, Egypt*

[d]*Stem Cell & Tissue Re-Engineering Program, Research Center, King Faisal Specialized Hospital & Research Center, MBC-03, PO Box 3354, Riyadh 11211, Saudi Arabia*

[e]*Department of Pharmacology and Toxicology, College of Pharmacy, King Saud University, Riyadh 11451, Saudi Arabia*

*Corresponding author:E-mail: mabhat@ksu.edu.sa; Phone: +966558164097

Experimental section

Chemistry

All chemicals and solvents were purchased with high purities and used without further purification. The progress of the reaction was monitored by thin layer chromatography (using silica gel 60 F-254 plates). The products were visualized with a 254 nm UV lamp. Melting points were determined by open capillary methods and are uncorrected. Products were purified by column chromatography on 100-200 mesh silica gel. The ^{1}H NMR spectra were recorded on 400 spectrometers using tetramethylsilane (TMS) as an internal standard. The ^{13}C NMR spectra were recorded at 100 MHz and chemical shifts were reported in parts per million (d) relative to tetramethylsilane (TMS) as an internal standard. Coupling constant (J) values were reported in hertz (Hz). The splitting patterns of the proton are described as s (singlet), d (doublet), dd (doublet of doublet), t (triplet), and m (multiplet) in ^{1}H NMR spectroscopic analysis. The products were confirmed by ^{1}H and ^{13}C NMR spectroscopy analysis. High-resolution mass spectra (HRMS) were obtained using Agilent 6520 (QTOF) ESI-HRMS model.

Experimental protocol for biological activity

Antitubercular testing using XRMA protocol:

All the chemicals such as sodium salt of XTT, DMSO, sulfanilic acid, sodium nitrate, HCl, NEED and rifampicin were purchased from Sigma-Aldrich, USA. Dubos medium was purchased from DIFCO, USA. Compounds were dissolved in DMSO and it was used as stock solution for further antimycobacterial testing. Microbial strains such as *MTB* H37Ra (ATCC 25177) and *M. bovis* BCG (ATCC 35734) were obtained from Astra-Zeneca, India. The stock culture was maintained at -80 ºC and sub-cultured once in a liquid medium before inoculation into an experimental culture. Cultures were grown in Dubos media (enrichment media). *Mycobacterium pheli* medium (minimal essential medium) was used for antimycobacterial assay. It contains 0.5 g KH_2PO_4, 0.25 g trisodium citrate, 60 mg $MgSO_4$, 0.5 g aspargine and 2 mL glycerol in distilled water (100 mL) followed by pH adjustment to 6.6. All the newly synthesized compounds were screened in vitro against two *Mycobacterium* species such as *MTB* H37Ra and *M. bovis* BCG. Both species of Mycobacterium were grown in *Mycobacterium pheli* medium. Screening of MTB H37Ra was done by using XTT reduction menadione assay (XRMA) and *M. bovis* BCG screening was done by using NR (Nitrate reductase) assay, both of them were developed.[1-2] Briefly 2.5 μL of these inhibitor solutions were added in a total volume of 250 μL of *Mycobacterium pheli* medium consisting of bacilli. The incubation was terminated on the 8th day for Active and 12th days for Dormant *MTB* culture. The XRMA and NR was then carried out to estimate viable cells present in different wells of the assay plate. The optical density was read on a micro plate reader (Spectramax plus384 plate reader, Molecular Devices Inc) at 470 nm filter for XTT and at 540 nm filter for NR against a blank prepared from cell-free wells. Absorbance given by cells treated with the vehicle alone was taken as 100% cell growth. Initially primary screening was done at 30, 10 and 3 μg/mL. Compounds showing 90% inhibition of bacilli at or lowers than 30 μg/mL were selected for further dose response curve. All experiments were performed in triplicates and the quantitative value was expressed as the average ± standard deviation. MIC and IC_{50} values of selected compound were calculated from their dose response

curves by using Origin 6 software. % Inhibition was calculated by using following formula: % Inhibition = [(absorbance of compound − absorbance of Test)/(absorbance of Control – absorbance of Blank)] × 100, where control is the medium with bacilli along with vehicle and blank is cell free medium.

Cytotoxic activity assay

Antiproliferative activity against MCF-7, A549, THP-1 and HCT-116 cell lines using the MTT assay:

Xanthene derivatives **3a-l** were assayed for their cytotoxic effects in three different cell lines, MCF-7, A549, THP-1 and HCT-116 using MTT assay.[3] The cell lines were maintained under standard cell culture conditions under 5% CO_2 at 37°C in 95% air humidified environment. Each concentration was tested in duplicates in a single experiment. GI_{50}/GI_{90} values were calculated using OriginPro Software.

Selectivity Index:

The SI was calculated by dividing the 50% growth inhibition concentration (GI_{50}) for cell lines (MCF-7, A549, THP-1 and HCT-116) by the MIC for in vitro activity against active/dormant *MTB* and BCG.[4]

Antibacterial activity:

All the bacterial cultures were first grown in Lysogeny Broth (LB) media at 37 °C at 180 RPM. Once the culture reaches 1 O.D, it was used for antibacterial assay. Bacterial strains *Escherichia coli* (NCIM-2688), *Pseudomonas fluorescens* (NCIM-2036) as Gram-negative and *Bacillus subtilis* (NCIM-2079), *Staphylococcus aureus* (NCIM-2010) as Gram-positive were obtained from NCIM (NCL, Pune) and were grown in Luria Burtony medium from Hi-media, India. The assay was performed in 96 well plates after 8 and 12 h for Gram negative and Gram positive bacteria, respectively. 0.1 % of 1 OD culture at 620 nm was used for screening.[5] 0.1 % inoculated culture was added in to each well of 96 well plates containing the compounds to be tested. Optical density for each plate was measured at 620 nm after 8 h for Gram negative bacteria and after 12 h for Gram positive bacteria.

References

1 Greenwood, D.; Slack, R.C.B.; Peutherer, J. F. *Medical Microbiology* 14th ed.; ELBS, London, **1992**.

2 (a) Singh, U.; Akhtar, S.; Mishra, A.; Sarkar, D. *J. Micro. Methods* **2011**, *84*, 202; (b) Khan, A.; Sarkar, S.; Sarkar, D. *Int. J. Antimicrob. Agents* **2008**, *32*, 40; (c) Sarkar, S.; Sarkar, D. *J. Biomol. Screen.* **2012**, *17*, 966.

3 Ciapetti, G.; Cenni, E.; Pratelli, L.; Pizzoferrato, A. *Biomaterials* **1993**, *14*, 359.

4 (a) Protopopova, M.; Hanrahan, C.; Nikonenko, B.; Samala, R.; Chen, P.; Gearhart, J.; Einck, L.; Nacy, C. A. *J. Antimicrob. Chemother.* **2005**, *56*, 968; (b) Poggi, M.; Barroso, R.; Costa-Filho, A. J.; de Barros, H. B.; Pavan, F.; Leite, C. Q.; Gambino, D.; Torre, M. H. *J. Mex. Chem. Soc.* **2013**, *57*,198; (c) Gundersen, L. L.; Meyer, J. N.; Spilsberg, B. *J. Med. Chem.* **2002**, *45*, 1383; (d) Sivakumar, K. K.; Rajasekaran, A. *Int. J. Drug Des. Disc.* **2013**, 1065.

5 Singh, R.; Nawale, L.U.; Arkile, M.; Shedbalkar, U. U.; Wadhwani, S. A.; Sarkar, D.; Chopade, B. A. *Int. J. Antimicrob. Agents* **2015**, *46*, 183.

2.3.1. 3,3,6,6-tetramethyl-9-phenyl-3,4,5,6,7,9-hexahydro-1H-xanthene-1,8(2H)-dione (3a)

The compound **3a** was obtained from condensation reaction **1a** and **2** as white solid; Mp: 205-206 °C; Yield: 93%; ^{1}H NMR (500 MHz, $CDCl_3$) δ 11.93 (s, 1H), 7.29-7.26 (t, *J* = 7.6 Hz, 2H), 7.19-7.09 (m, 3H), 5.56 (d, *J* = 60.1 Hz, 1H), 2.49-2.30 (m, 9H), 1.24-1.11 (dd, *J* = 64.4, 29.0 Hz, 12H); ^{13}C NMR (101 MHz, $CDCl_3$) δ 195.65 (C=O), 164.07 (C=C-O), 143.16 (Ar-H), 135.05 (Ar-H), 129.49 (Ar-H), 127.51 (Ar-H), 119.84 (Ar-H), 50.45 (CH), 42.23 (CH_2), 32.45 (CH_2), 31.34 (CH_2), 29.56 (CH_3) and 29.33 (CH_3); HRMS (ESI-qTOF): Calcd for $C_{23}H_{27}O_3$ $[M+H]^+$, 351.0920: found: 351.0903.

2.3.2. 3,3,6,6-tetramethyl-9-(m-tolyl)-3,4,5,6,7,9-hexahydro-1H-xanthene-1,8(2H)-dione (3b)

The compound **3b** was obtained from condensation reaction **1a** and **2** as white solid; Mp: 208-210 °C; Yield: 86%; ^{1}H NMR (400 MHz, $CDCl_3$) δ 7.10-6.99 (m, 3H), 6.86 (d, *J* = 6.8 Hz, 1H), 4.68 (s, 1H), 2.44 (s, 4H), 2.24 (s, 3H), 2.20-2.10 (t, *J* = 13.8 Hz, 4H), 1.05 (s, 6H), 0.95 (s, 6H); ^{13}C NMR (101 MHz, $CDCl_3$) δ 196.31 (C=O), 162.23 (C=C-O), 144.01 (Ar-H), 137.21 (Ar-H), 129.38 (Ar-H), 127.84 (Ar-H), 127.13 (Ar-H), 125.21 (Ar-H), 115.67 (Ar-H), 50.76 (CH), 40.84 (CH_2), 32.17 (CH_2), 31.68 (CH_2), 29.24 (CH_2), 27.30 (CH_3) and 21.48 (CH_3); HRMS (ESI-qTOF): Calcd for $C_{24}H_{29}O_3$ $[M+H]^+$, 365.0650: found: 365.0673.

2.3.3. 3,3,6,6-tetramethyl-9-(p-tolyl)-3,4,5,6,7,9-hexahydro-1H-xanthene-1,8(2H)-dione (3c)

The compound **3c** was obtained from condensation reaction **1c** and **2** as yellow solid; Mp: 222-224 °C; Yield: 90%; ^{1}H NMR (400 MHz, $CDCl_3$) δ 7.15-7.14 (d, J = 7.8 Hz, 2H), 6.99-6.97 (d, J = 7.5 Hz, 2H), 4.68 (s, 1H), 2.43 (s, 4H), 2.20 (s, 3H), 2.17-2.09 (m, 4H), 1.06 (s, 6H), 0.95 (s, 6H); ^{13}C NMR (101 MHz, $CDCl_3$) δ 196.36 (C=O), 162.17 (C=C-O), 141.21(Ar-C), 135.61(Ar-C), 128.71 (Ar-C), 128.20 (Ar-C), 115.67 (Ar-C), 50.75 (CH), 40.81 (CH_2), 32.13 (CH_2), 31.40 (CH_2), 29.23 (CH_2), 27.31 (CH_3) and 21.02 (CH_3); HRMS (ESI-qTOF): Calcd for $C_{24}H_{29}O_3$ $[M+H]^+$, 365.5041: found: 365.5012.

2.3.4. 9-(3-methoxyphenyl)-3,3,6,6-tetramethyl-3,4,5,6,7,9-hexahydro-1H-xanthene-1,8(2H)-dione (3d)

The compound **3d** was obtained from condensation reaction **1d** and **2** as yellow solid; Mp: 162-164 °C; Yield: 84%; ^{1}H NMR (400 MHz, $CDCl_3$) δ 7.05-7.01 (t, J = 8.1 Hz, 1H), 6.80-6.79 (d, J = 6.9 Hz, 2H), 6.57-6.55 (d, J = 7.0 Hz, 1H), 4.66 (s, 1H), 3.66 (s, 3H), 2.40 (s, 4H), 2.17-2.06 (q, J = 16.2 Hz, 4H), 1.01 (s, 6H), 0.91 (s, 6H); ^{13}C NMR (101 MHz, $CDCl_3$) δ 196.26 (C=O), 162.35 (C=C-O), 159.26 (Ar-C), 145.68 (Ar-C), 128.77 (Ar-C), 120.72 (Ar-C), 115.42 (Ar-C), 114.26 (Ar-C), 111.78 (Ar-C), 55.01(OCH_3), 50.70 (CH), 40.75 (CH_2), 32.09 (CH_2), 31.73 (CH_2), 29.18 (CH_3) and 27.29 (CH_3); HRMS (ESI-qTOF): Calcd for $C_{24}H_{29}O_4$ $[M+H]^+$, 381.1631: found: 381.1645.

2.3.5. 9-(4-methoxyphenyl)-3,3,6,6-tetramethyl-3,4,5,6,7,9-hexahydro-1H-xanthene-1,8(2H)-dione (3e)

The compound **3e** was obtained from condensation reaction **1e** and **2** as yellow solid; Mp: 248-250 °C; Yield: 90%; ^{1}H NMR (400 MHz, $CDCl_3$) δ 7.17-7.15 (m, 2H), 6.71-6.69 (dd, J = 5.8, 2.6 Hz, 2H), 4.65 (s, 1H), 3.68 (d, J = 4.9 Hz, 3H), 2.42 (s, 4H), 2.20 – 2.09 (m, 4H), 1.05 (s, 6H), 0.94 (s, 6H); ^{13}C NMR (101 MHz, $CDCl_3$) δ 196.40 (C=O), 162.05 (C=C-O), 157.90 (Ar-C), 136.47 (Ar-C), 129.25 (Ar-C), 115.72 (Ar-C), 113.41 (Ar-C), 55.05 (CH_3), 50.74 (CH), 40.81 (CH_2), 32.14 (CH_2), 30.92 (CH_2), 29.23 (CH_3) and 27.29 (CH_3); HRMS (ESI-qTOF): Calcd for $C_{24}H_{29}O_4$ $[M+H]^+$, 381.1150: found: 381.1132.

2.3.6. 9-(3,4-dimethoxyphenyl)-3,3,6,6-tetramethyl-3,4,5,6,7,9-hexahydro-1H-xanthene-1,8(2H)-dione (3f)

The compound **3f** was obtained from condensation reaction **1f** and **2** as yellow solid; Mp: 200-202 °C; Yield: 89%; ^{1}H NMR (400 MHz, $CDCl_3$) δ 6.88 (s, 1H), 6.75-6.68 (td, J = 8.2, 4.3 Hz, 2H), 4.68 (s, 1H), 3.83 (d, J = 1.8 Hz, 3H), 3.77 (d, J = 1.7 Hz, 3H), 2.44 (s, 4H), 2.24 –

2.14 (m, 4H), 1.09 (s, 6H), 0.98 (s, 6H); ^{13}C NMR (101 MHz, $CDCl_3$) δ 196.42 (C=O), 162.08 (C=C-O), 148.44 (Ar-C), 147.45 (Ar-C), 136.98 (Ar-C), 120.09 (Ar-C), 115.73 (Ar-C), 112.31 (Ar-C), 110.85 (Ar-C), 55.85 (CH_3), 50.74 (CH), 40.87 (CH_2), 32.15 (CH_2), 31.20 (CH_2), 29.30 (CH_3) and 27.14 (CH_3); HRMS (ESI-qTOF): Calcd for $C_{25}H_{31}O_5$ $[M+H]^+$, 411.2860: found: 411.2845.

2.3.7. 3,3,6,6-tetramethyl-9-(3-nitrophenyl)-3,4,5,6,7,9-hexahydro-1H-xanthene-1,8(2H)-dione (3g)

The compound **3g** was obtained from condensation reaction **1g** and **2** as red solid; Mp: 172-174 ºC; Yield: 82%; ^{1}H NMR (400 MHz, $CDCl_3$) δ 8.01 – 7.95 (m, 2H), 7.79-7.77 (d, *J* = 6.7 Hz, 1H), 7.40-7.38 (d, *J* = 7.8 Hz, 1H), 4.81 (s, 1H), 2.49 (s, 4H), 2.25-2.12 (q, *J* = 16.3 Hz, 4H), 1.09 (s, 6H), 0.97 (s, 6H), ^{13}C NMR (101 MHz, $CDCl_3$) δ 196.30 (C=O), 162.98 (C=C-O), 148.32 (Ar-C), 146.26 (Ar-C), 135.72 (Ar-C), 128.76 (Ar-C), 112.48 (Ar-C), 121.64 (Ar-C), 114.53 (Ar-C), 50.62 (CH), 40.81 (CH_2), 32.22 (CH_2), 32.07 (CH_2), 29.17 (CH_3)and 27.29 (CH_3); HRMS (ESI-qTOF): Calcd for $C_{23}H_{26}NO_5$ $[M+H]^+$, 396.3451: found: 396.3469.

2.3.8. 9-(3-iodophenyl)-3,3,6,6-tetramethyl-3,4,5,6,7,9-hexahydro-1H-xanthene-1,8(2H)-dione (3h)

The compound **3h** was obtained from condensation reaction **1h** and **2** as pale yellow solid; Mp: 276-278 ºC; Yield: 84%; ^{1}H NMR (400 MHz, $CDCl_3$) δ 7.53 (s, 1H), 7.42-7.41 (d, *J* = 7.8 Hz, 1H), 7.32-7.24 (d, *J* = 7.6 Hz, 1H), 6.96-6.92 (t, *J* = 7.8 Hz, 1H), 4.60 (s, 1H), 2.45 (s, 4H), 2.24-2.14 (m, 4H), 1.08 (s, 6H), 0.99 (s, 6H); ^{13}C NMR (101 MHz, $CDCl_3$) δ 196.21 (C=O), 162.48 (C=C-O), 146.36 (Ar-C), 137.07 (Ar-C), 135.48 (Ar-C), 129.76 (Ar-C), 128.20 (Ar-C), 115.05 (Ar-C), 94.07 (Ar-C), 50.67 (CH), 40.84 (CH_2), 32.21 (CH_2), 31.61 (CH_2), 29.67 (CH_2), 29.20 (CH_3) and 27.32 (CH_3); HRMS (ESI-qTOF): Calcd for $C_{23}H_{26}IO_3$ $[M+H]^+$, 477.3570: found: 477.3526.

2.3.9. 9-(4-bromophenyl)-3,3,6,6-tetramethyl-3,4,5,6,7,9-hexahydro-1H-xanthene-1,8(2H)-dione (3i)

The compound **3i** was obtained from condensation reaction **1i** and **2** as red solid; Mp: 238-240 ºC; Yield: 86%; ^{1}H NMR (400 MHz, $CDCl_3$) δ 7.34-7.32 (d, *J* = 6.9 Hz, 2H), 7.18-7.16 (d, *J* = 6.8 Hz, 2H), 4.69 (s, 1H), 2.46 (s, 4H), 2.25-2.14 (q, *J* = 16.4 Hz, 4H), 1.10 (s, 6H), 0.98 (s, 6H); ^{13}C NMR (101 MHz, $CDCl_3$) δ 196.26 (C=O), 162.41 (C=C-O), 143.18 (Ar-C), 131.09 (Ar-C), 130.13 (Ar-C), 120.18 (Ar-C), 115.14 (Ar-C), 50.65 (CH), 40.81 (CH_2), 32.15

(CH_2), 31.52 (CH_2), 29.21 (CH_3) and 27.26 (CH_3); HRMS (ESI-qTOF): Calcd for $C_{23}H_{26}BrO_3$ $[M+H]^+$, 430.2450: found: 430.2400.

2.3.10. **9-(4-chlorophenyl)-3,3,6,6-tetramethyl-3,4,5,6,7,9-hexahydro-*1H*-xanthene-1,8(*2H*)-dione (3j)**

The compound **3j** was obtained from condensation reaction **1j** and **2** as yellow solid; Mp: 238-240 ºC; Yield: 90%; ^1H NMR (400 MHz, $CDCl_3$) δ 7.24 (d, J = 8.4 Hz, 2H), 7.21 – 7.14 (m, 2H), 4.71 (s, 1H), 2.47 (s, 4H), 2.19 (q, J = 16.3 Hz, 4H), 1.10 (s, 6H), 0.98 (s, 6H); ^{13}C NMR (101 MHz, $CDCl_3$) δ 196.28 (C=O), 162.44 (C=C-O), 142.70 (Ar-C), 131.93 (Ar-C), 129.74 (Ar-C), 128.13 (Ar-C), 115.18 (Ar-C), 50.66 (CH), 40.79 (CH_2), 32.14 (CH_2), 31.43 (CH_2), 29.21 (CH_3) and 27.24 (CH_3); HRMS (ESI-qTOF): Calcd for $C_{23}H_{26}ClO_3$ $[M+H]^+$, 385.1165: found: 385.1129.

2.3.11. **9-(4-hydroxyphenyl)-3,3,6,6-tetramethyl-3,4,5,6,7,9-hexahydro-*1H*-xanthene-1,8(*2H*)-dione (3l)**

The compound **3l** was obtained from condensation reaction **1l** and **2** as red solid; Mp: 248-250 ºC; Yield: 83%; ^1H NMR (400 MHz, $CDCl_3$) δ 7.31 (s, 1H), 7.07-7.05 (d, J = 7.8 Hz, 2H), 6.55-6.53 (d, J = 7.8 Hz, 2H), 4.66 (s, 1H), 2.46 (s, 4H), 2.26-2.16 (q, J = 16.4 Hz, 4H), 1.08 (s, 6H), 0.99 (s, 6H). ^{13}C NMR (101 MHz, $CDCl_3$) δ 197.30 (C=O), 162.45 (C=C-O), 154.70 (Ar-C), 135.47 (Ar-C), 129.29 (Ar-C), 115.85 (Ar-C), 115.23 (Ar-C), 50.72 (CH_2), 40.81 (CH_2), 32.23 (CH_2), 30.93 (CH_2), 29.11 (CH_3) and 27.35 (CH_3); HRMS (ESI-qTOF): Calcd for $C_{23}H_{27}O_4$ $[M+H]^+$, 367.0652: fund: 367.0673.

2.3.12. **9-cyclohexyl-3,3,6,6-tetramethyl-3,4,5,6,7,9-hexahydro-*1H*-xanthene-1,8(*2H*)-dione (3m)**

The compound **3m** was obtained from condensation reaction **1m** and **2** as white solid; Mp: 176-178 ºC; Yield: 82%; ^1H NMR (400 MHz, $CDCl_3$) δ 7.67 (s, 2H), 5.46 (s, 1H), 3.31 (s, 2H), 2.50 (s, 4H), 2.23 (s, 4H), 1.22 (s, 1H), 1.06-0.85 (d, J = 18.0 Hz, 12H); ^{13}C NMR (101 MHz, $CDCl_3$) δ 193.30 (C=O), 165.54 (C=C-O), 111.77 (Ar-C), 50.43 (CH), 40.71 (CH_2), 34.51 (CH_2), 33.01 (CH_2), 31.25 (CH_2), 29.20 (CH_2), 27.42 (CH_2), 25.93 (CH_2), 24.52 (Cy-C), 23.21 (Cy-C), 23.15 (CH_3) and 23.07 (CH_3); HRMS (ESI-qTOF): Calcd for $C_{23}H_{33}O_3$ $[M+H]^+$, 357.1081: found: 357.1064.

^{1}H and ^{13}C NMR of Representative Compounds

3a. ^{1}H NMR, 400 MHz, DMSO-d_6

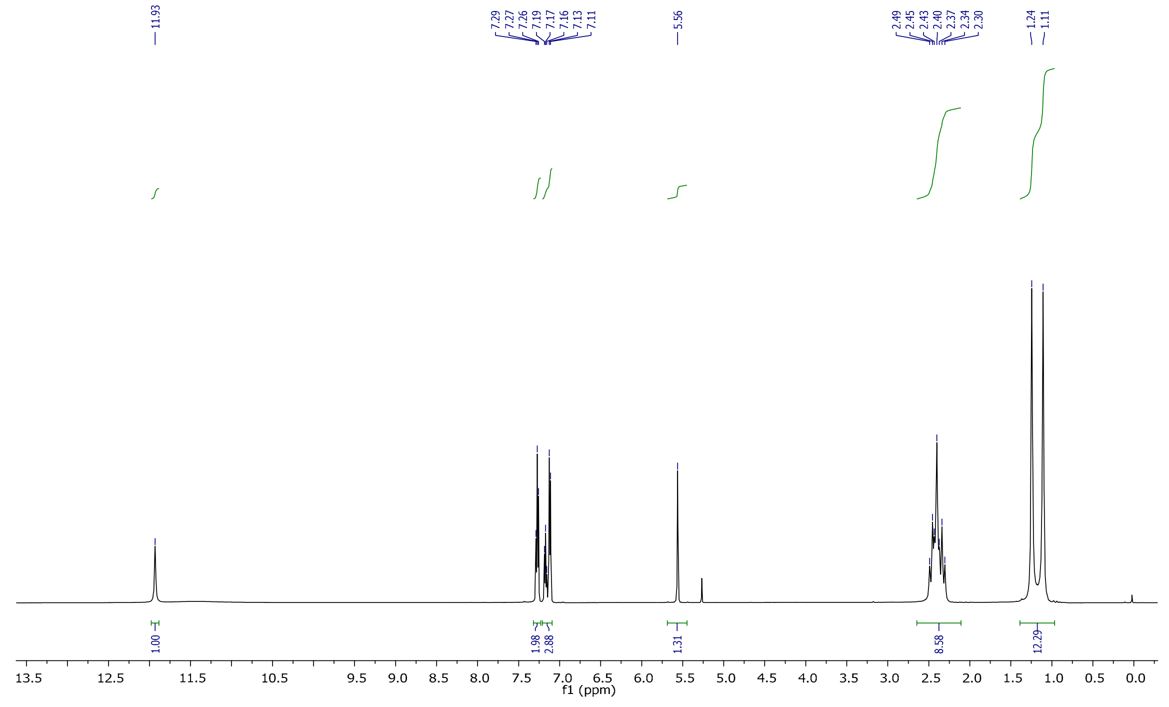

3a. ^{13}C NMR, 100 MHz, DMSO-d_6

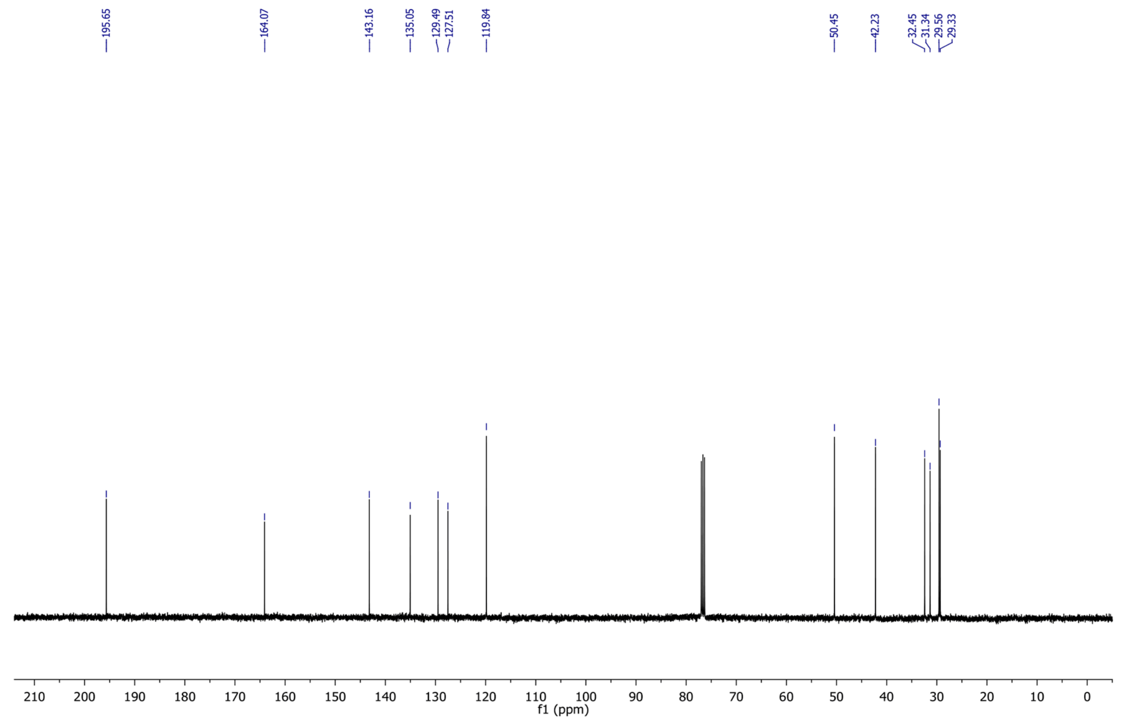

Supplement: Supplementary file 1 [file molecules-26-03667-s001.zip › molecules-1076108-SI.pdf]
